# Potential Protective Effect of Dengue NS1 Human Monoclonal Antibodies against Dengue and Zika Virus Infections

**DOI:** 10.3390/biomedicines11010227

**Published:** 2023-01-16

**Authors:** Rochanawan Sootichote, Wilarat Puangmanee, Surachet Benjathummarak, Siriporn Kowaboot, Atsushi Yamanaka, Korbporn Boonnak, Sumate Ampawong, Supawat Chatchen, Pongrama Ramasoota, Pannamthip Pitaksajjakul

**Affiliations:** 1 Department of Social and Environmental Medicine, Faculty of Tropical Medicine, Mahidol University, Bangkok 10400, Thailand; 2 Center of Excellence for Antibody Research, Faculty of Tropical Medicine, Mahidol University, Bangkok 10400, Thailand; 3 Faculty of Medical Technology, Rangsit University, Pathumthani 12000, Thailand; 4 Research Institute for Microbial Diseases, Osaka University, Osaka 565-0871, Japan; 5 Department of Immunology, Faculty of Medicine, Siriraj Hospital, Mahidol University, Bangkok 10700, Thailand; 6 Department of Tropical Pathology, Faculty of Tropical Medicine, Mahidol University, Bangkok 10400, Thailand; 7 Department of Tropical Pediatrics, Faculty of Tropical Medicine, Mahidol University, Bangkok 10400, Thailand

**Keywords:** dengue virus, Zika virus, dengue non-structural protein 1, human monoclonal antibody, neutralization, vascular leakage, therapeutic antibody

## Abstract

Due to the lack of an effective therapeutic treatment to flavivirus, dengue virus (DENV) nonstructural protein 1 (NS1) has been considered to develop a vaccine owing to its lack of a role in antibody-dependent enhancement (ADE). However, both NS1 and its antibody have shown cross-reactivity to host molecules and have stimulated anti-DENV NS1 antibody-mediated endothelial damage and platelet dysfunction. To overcome the pathogenic events and reactogenicity, human monoclonal antibodies (HuMAbs) against DENV NS1 were generated from DENV-infected patients. Herein, the four DENV NS1-specific HuMAbs revealed the therapeutic effects in viral neutralization, reduction of viral replication, and enhancement of cell cytolysis of DENV and zika virus (ZIKV) via complement pathway. Furthermore, we demonstrate that DENV and ZIKV NS1 trigger endothelial dysfunction, leading to vascular permeability in vitro. Nevertheless, the pathogenic effects from NS1 were impeded by 2 HuMAbs (D25-4D4C3 and D25-2B11E7) and also protected the massive cytokines stimulation (interleukin [IL-]-1b, IL-1ra, IL-2, IL-4, IL-5, IL-6, IL-8, IL-9, IL-13, IL-17, eotaxin, granulocyte colony-stimulating factor, granulocyte-macrophage colony-stimulating factor, Inducible protein-10, monocyte chemoattractant protein-1, macrophage inflammatory protein [MIP]-1 α, MIP-1β, tumor necrosis factor-α, platelet-derived growth factor, and RANTES). Collectively, our findings suggest that the novel protective NS1 monoclonal antibodies generated from humans has multiple therapeutic benefits against DENV and ZIKV infections.

## 1. Introduction

Dengue virus (DENV) and zika virus (ZIKV) are mosquito-borne viral infections that occur worldwide. They belong to the *Flavivirus* genus and Flaviviridae family. There are four dengue serotypes (DENV 1–DENV 4), which are antigenically distinguishable. The global burden of the disease has increased annually, with estimated tens of millions of cases of dengue fever (DF), including up to 500,000 cases of life-threatening dengue hemorrhagic fever/dengue shock syndrome (DHF/DSS) [1]. In 2009, the World Health Organization classified dengue disease into dengue without warning signs, dengue with warning signs (i.e., abdominal pain/ tenderness, persistent vomiting, clinical fluid accumulation, mucosal bleed, lethargy, liver enlargement >2 cm, and increase in hematocrit concurrent with a rapid decrease in the platelet count), and severe dengue. ZIKV infection causes severe birth defects (termed congenital Zika syndrome) and Guillain-Barré syndrome in adults [2,3]. Notably, ZIKV re-emergence was reported for all areas with prior reports of viral transmission [4].

DENV and ZIKV are positive-sense RNA viruses with genomes of approximately 11 kb. The genome is translated into a single polyprotein, which is subsequently cleaved and processed by host and viral proteases into three structural proteins, a capsid (C), membrane (M), and envelope (E) protein, as well as seven nonstructural proteins (NS1, NS2A, NS2B, NS3, NS4A, NS4B, and NS5) [5].

To date, only supportive treatment is available because the mechanisms leading to severe disease caused by these viruses are poorly understood, There is no effective licensed vaccine available for DENV and ZIKV infections. Only one licensed dengue vaccine has been introduced in endemic countries; however, the quality, safety, and efficacy of the vaccine remain unclear [6].

NS1, an immature monomeric nonstructural protein in the endoplasmic reticulum, is processed into a stable homodimeric form that can be covalently linked to the surface membrane through glycosylphosphatidylinositol [7]. Moreover, NS1 is a highly conserved protein among flaviviruses with 20–40% identity and 60–80% similarity, consisting of 352 amino acids with a molecular weight of 46–55 kDa depending on its glycosylation status [8,9]. NS1 is a multifunctional protein: intracellular NS1 is important for viral replication and assembly, whereas secreted NS1 leaves infected cells, circulates in the host’s bloodstream [8], and is associated with immune evasion [10,11].

During the pathogenesis of DENV infection, antibody-dependent enhancement (ADE) is a major cause of severe disease progression. However, ADE does not explain why vascular leakage and hemorrhage occur in patients with DHF/DSS. NS1 is of interest for pathogenesis studies and DENV vaccine strategies. Complement activation by DENV NS1 leads to complement-mediated lysis and vascular leakage in patients with DHF/DSS [12]. Moreover, NS1 mimics the characteristic features of lipopolysaccharides via toll-like receptor (TLR) 4 and triggers cytokine secretion and platelet activation, as well as enhances aggregation leading to thrombocytopenia and vascular leakage [12,13,14]. Furthermore, DENV NS1 mediates endothelial dysfunction and hyperpermeability by disrupting the endothelial glycocalyx [15]. Additionally, macrophage migration inhibitory factor was associated with DENV NS1-induced glycocalyx degradation and vascular leakage, which may enhance dengue severity [16]. In vitro and in vivo studies showed that NS1 is a virulent factor involved in hemorrhage, thrombocytopenia, and coagulopathy, which are related to severe dengue disease. Based on the structure of NS1, NS1 antigens and anti-NS1 antibodies do not cause ADE; however, they can cross-react with host proteins, including human plasminogen, thrombin, platelets, and endothelial cells, and thereby play a significant role in the pathogenesis of severe dengue disease [17]. Modified NS1 antigen or antibodies against modified NS1 without cross-reactivity to host antigens provided mice with effective protection against DENV infection with reduced bleeding time and hemorrhage [18,19].

In this study, we investigated the different characteristics of four DENV NS1-specific human antibodies generated from hybridoma clones as a potential therapeutic agent. The effects of NS1 human monoclonal antibodies (HuMAbs) against viral replication and cytolysis via complement pathway, endothelial permeability, and cytokine release in vitro were tested. Additionally, epitopes were identified and characterized. This study highlights the potential use of anti-NS1 HuMAbs as a candidate therapeutic target against DENV and ZIKV infections.

## 2. Materials and Methods

### 2.1. Cell Cultures

A human microvascular endothelial cell line (HMEC-1) was grown in adherent cell culture using endothelial cell growth medium (MCDB131) (Merck, Branchburg, NJ, USA) supplemented with 10% heat-inactivated fetal bovine serum (FBS) (HyClone, Logan, UT, USA), 0.1 mg/mL of hydrocortisone, 1 μg/mL of epidermal growth factor, and 200 mM of L-glutamine. Human umbilical vein endothelial cells (HUVECs) were grown in F-12K medium (ATCC 30-2004) supplemented with 10% heat-inactivated FBS, 0.1 mg/mL of heparin, and 30 µg/mL of endothelial cell growth supplement. Vero cells (ATCC^®^ CRL. 1586™) are a cell line derived from kidney epithelial cells from American green monkeys (*Cerfcopithecus aethiopsi*). Vero cells were cultured in minimum essential medium with Earle’s balanced salts (HyClone, Logan, UT, USA) supplemented with 10% heat-inactivated FBS. The cells were sub-passaged to correct confluency every 3–5 days by 0.25% trypsin/EDTA and grown at 37 °C in 5% CO_2_ atmosphere. Trypan blue staining was used to measure viable cells using a hematocytometer under microscopy. Cell was stored at −80 °C in cell banker (ZENOAQ, Tokyo, Japan) as a storage medium.

### 2.2. Virus Cultures

DENV1 (Mochizuki strain), DENV2 (New Guinea C or NGC strain), DENV3 (H87 strain), DENV4 (H241 strain), and ZIKV (SV0127/14 strain) were propagated in C6/36 cells. Briefly, a monolayer of C6/36 cells was cultured with DENV or ZIKV at a multiplicity of infection (MOI) of 0.1 and incubated at 28 °C for 5–7 days (for DENV) or 10 days (for ZIKV). The culture supernatant containing viruses was collected and clarified by centrifugation at 1000× *g* for 20 min at 4 °C. The supernatant was aliquoted and stored as virus stock at −80 °C until use. Infectivity titers were estimated according to the number of focus forming units (FFUs) on Vero cells.

### 2.3. Preparation of MAbs

Anti-DENV NS1 HuMAbs were generated from hybridoma cells previously produced at the Center of Excellence for Antibody Research (Tropical Medicine, Mahidol University). Based on the hybridoma technique, anti-NS1 HuMAbs were generated from lymphocytes obtained from patients with secondary dengue infection at the convalescent phase [20]. In this study, we selected four anti-NS1 HuMAbs (D25-2B11C3 [M1], D25-4D4C3 [M20], D26-5A2G2 [238], D25-2B11E7 [8]). These four HuMAbs were derived from two Thai patients with dengue at the Hospital for Tropical Diseases, Faculty of Tropical Medicine, Mahidol University (Table 1) [20]. All hybridomas were cultured at 37 °C, in a humidified 5% CO_2_ incubator in Dulbecco’s Modified Eagle Medium (DMEM) (Gibco, Thermo fisher Scientific, Waltham, MA, USA) supplemented with 10–15% heat-inactivated FBS. Antibody-secreting hybridomas were re-cloned by limiting dilution to obtain a single clone-producing NS1 antibody. The secreted DENV NS1 HuMAbs were screened by immunofluorescence assay. Subsequently, positive hybridomas were expanded, and culture fluid was collected from each hybridoma. Secreted HuMAbs were purified and detected using a protein A column (HiTrap MabSelect SuRe, GE Healthcare, Freiburg, Germany) and Western blot analysis, respectively.

### 2.4. Neutralization Assay

HMEC-1 cells and HUVECs were seeded into 96-well plates (1 × 10^4^ cells/well) and infected with DENV or ZIKV (MOI = 0.125), respectively, for 1 h at 37 °C. After 1 h of incubation, the residual virus was removed using PBS, and the infected cells were incubated with 100 μL/well of various concentrations of antibodies and further incubated at 37 °C in a 5% CO_2_ incubator. For further determination of virus titer, culture fluids were subsequently collected at 24 and 48 h post-infection.

### 2.5. DENV NS1 HuMAbs and Complement-Affected DENV Replication

HMEC-1 cells and HUVECs in 96-well plates were infected with DENV and ZIKV (MOI = 5), respectively, for 1 h at 37 °C. The residual extracellular virus was removed by washing three times with 100 μL of PBS, and a new complete medium was added for 12 h. After 12 h of incubation, the medium was replaced with 2% FBS culture medium containing NS1 HuMAbs (100 µg/mL) and Low-Tox-M rabbit complement at 1:20 dilution (Cedarlane Laboratories Ltd., Birlington, ON, Canada) for an additional 36 h incubation at 37 °C in a 5% CO_2_ incubator. At 12 h intervals, culture supernatants were collected to measure virus titers by focus forming unit per ml (FFU/mL).

### 2.6. Fluorescent Focus Assay

In brief, Vero cells were seeded into 96-well plates to obtain a cell monolayer on the next day. Culture supernatants at different time points after infection were inoculated onto the cells and incubated for 2 h at 37 °C, which were subsequently overlaid with MEM medium containing 2% carboxymethyl cellulose and 3% FBS. Cells were incubated at 37 °C for 3 days. Then, cells were fixed with 3.7% formaldehyde in PBS for 20 min and permeabilized by 0.1% Triton X-100 in PBS for 5 min at room temperature. Virus foci were stained with anti-E HuMAb, followed by Alexa Fluor 488 goat anti-human IgG (H + L) antibody (1:1000) (Invitrogen, Waltham, MA, USA) and visualized using a fluorescent microscope (Olympus IX71, Tokyo, Japan) to determine the FFU.

### 2.7. Antibody-Dependent Complement-Mediated Cytolytic Assay

HMEC-1 and HUVECs were inoculated with DENV and ZIKV (MOI = 5), respectively, at 37 °C for 1 h. Following centrifugation to remove residual virus in the culture supernatant, cells were cultured into 96-well tissue-culture plates (1 × 10^4^ cells/well) at 37 °C in 5% CO_2_. Virus-infected cells (approximately 45% E antigen-positive) were harvested after 36 h incubation and incubated with HuMAbs (100 µg/mL) at 37 °C for 30 min. After washing twice with PBS, cells were incubated with Low-Tox-M rabbit complement (1:20) (Cedarlane Laboratories Ltd., Birlington, ON, Canada) at 37 °C for 4 h in a 5% CO_2_ incubator to facilitate complement-mediated cell lysis. Cytolysis was determined by the release of lactate dehydrogenase (LDH) as a cytoplasmic enzyme using a commercial kit (CytoTox96^®^ Non-Radioactive Cytotoxicity Assay, Promega, Madison, WI, USA). The optical density at a 490 nm absorbance wavelength was measured using a microplate reader.

### 2.8. Vascular Permeability Assay

To evaluate the disruptive effect of DENV NS1 HuMAbs on flavivirus NS1-triggered vascular permeability, HMEC-1 and HUVECs were cultured in a 24-well Transwell polycarbonate membrane system (Transwell permeable support, 0.4 µM, 6.5 mm Transwell insert, Corning, NY, USA) for 5 days. Subsequently, the medium was replaced in both the upper and lower chambers. The cell monolayer was incubated with either NS1 protein (10 µg/mL) to disturb the vascular integrity as positive control or NS1 with each HuMAb (100 µg/mL). Untreated cells-containing Transwell inserts were used as a negative control. After 24 h stimulation, the medium was replaced in the upper and lower chambers. Vascular permeability was examined by refilling the top chamber with medium containing streptavidin-HRP (ab7403, Abcam, Cambridge, UK). After 5 min incubation, culture fluid from the lower chamber was collected, and HRP activity was visualized by color development after adding the TMB substrate and 2N H_2_SO_4_ as a stop solution. The absorption at OD 450 nm was acquired with an ELISA reader (BioTek Synergy H1 Microplate reader, Santa Clara, CA, USA). The relative permeability of all tests was calculated by normalizing with a non-treated cell.

### 2.9. Cytokine and Chemokine Analyses

Culture supernatants from NS1-treated, HuMAbs-treated, NS1/HuMAbs-treated, and complete media-treated HMEC-1 and HUVECs used in the vascular permeability assay were used to measure the levels of 27 cytokines including interleukin (IL)-1β, IL-1rα, IL-2, IL-4, IL-5, IL-6, IL-7, IL-8, IL-9, IL-10, IL-12 (p70), IL-13, IL-15, IL17, granulocyte colony-stimulating factor (G-CSF), granulocyte macrophage colony-stimulating factor (GM-CSF), interferon (IFN)-γ, IFN-γ-inducible protein (IP)-10, monocyte chemoattractant protein (MCP)-1, macrophage inflammatory protein (MIP)-1β, MIP-1α, eotaxin, basic-fibroblast growth factor (basic-FGF), platelet-derived growth factor (PDGF), vascular endothelial growth factor (VEGF), RANTES, and tumor necrosis factor (TNF)-α using a Bio-Plex Pro Human Cytokine 27-plex assay and a Luminex machine (Bio-Rad, Hercules, CA, USA) according to the manufacturer’s instructions. Supernatants were analyzed using the standard controls run in duplicate.

### 2.10. Plasmid Construction and Transfection

A plasmid, which expresses epitopes of the DENV NS1 protein, was constructed by cloning. Initially, DENV NS1 cDNAs were amplified by PCR with different sets of primers (Table 2) that overlapped the following three distinct domains of NS1: the β-roll (residues 1–29), the wing (residues 30–180), and the β-ladder (residues 181–352). The PCR products were inserted into the *Xho*I/*BamH*I sites of a pET32b expression vector (Sigma, St. Louis, MO, USA) and subsequently transformed into *Escherichia coli* (*E. coli*). The sets of truncated plasmids were expressed in *E. coli* strain BL21-DE3 via isopropyl β-D-1-thiogalactopyranoside induction. According to epitope mapping, *E. coli* BL21-DE3 expressing truncated NS1 proteins were lysed in a sodium-dodecyl-sulfate polyacrylamide gel electrophoresis (SDS-PAGE) sample buffer in the presence of β-mercaptoethanol and subsequently heated at 100 °C for 10 min. Proteins were separated on 10% SDS-PAGE gels and transferred to polyvinylidene difluoride (PVDF) membranes. The non-specific binding of membranes was blocked with 5% skim milk in Tris-buffered saline (TBS) containing 0.05% Tween 20. Membranes were subsequently incubated overnight with HuMAbs. After washing with TBS-T, the membranes were incubated with horseradish peroxidase (HRP)-conjugated anti-human IgG (H + L) (1:10,000) (Merck, Branchburg, NJ, USA) for 1 h and visualized using ECL Western blotting detection reagent (GE Healthcare, Freiburg, Germany).

### 2.11. Analysis of NS1 Sequences and Amino Acid Variation within Epitope Regions

Overall, 1228 DENV1 sequences, 815 DENV2 sequences, 572 DENV3 sequences, and 91 DENV4 sequences were collected from the National Center of Biotechnology Information protein database. The amino acid sequences within epitope regions were analyzed using Bioedit version 7 (Ibis Biosciences, Abbott, Chicago, IL, USA). Variations in each amino acid residue were displayed on an Entropy H(x) plot.

### 2.12. Statistical Analysis

Data were presented as mean ± standard deviation (SDs). Comparisons between groups of treatments were determined by the Student’s *t*-test and two-way ANOVA with Tukey’s test. Statistical analyses were performed using GraphPad Prism 8 software, and all graphs were generated using Prism 8. *p*-values < 0.05 were considered statistical significance.

## 3. Results

### 3.1. DENV NS1 HuMAbs Neutralize Flaviviruses

**Figure 1:**
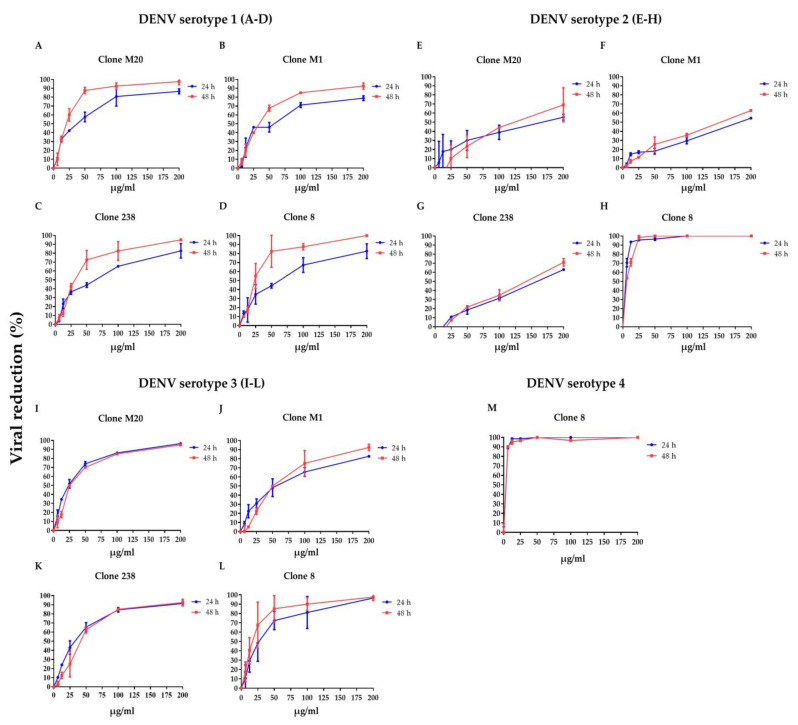
Anti-DENV NS1 HuMAbs induce DENV neutralization in endothelial cells: HMEC-1 were infected with four DENV serotypes (MOI = 0.125): (**A**–**D**) DENV 1, (**E**–**H**) DENV 2, (**I**–**L**) DENV 3, and (**M**) DENV 4 at 24 and 48 h post-infection. To determine the neutralization potency represented by the percentage of viral reduction in Vero cells, culture supernatant from four anti-DENV NS1 HuMAb (M20, M1, 238, 8)-treated infected cells are collected. The results are presented as the mean ± SD of duplicate independent experiments.

**Figure 2:**
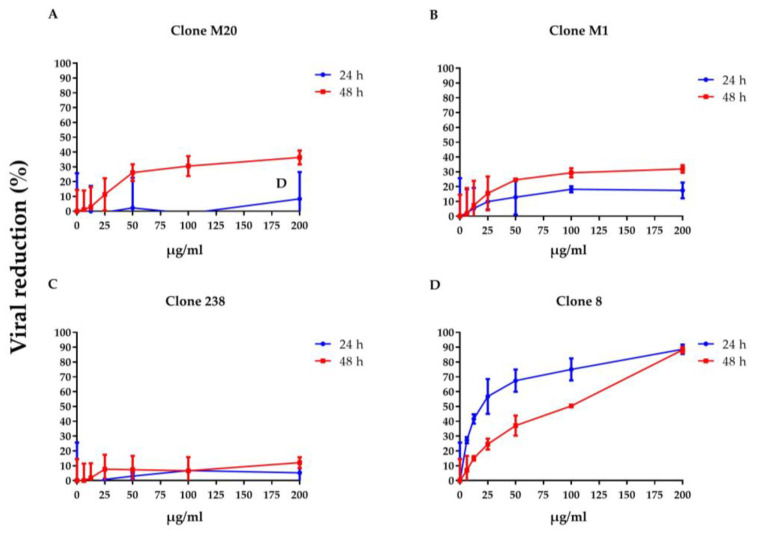
Anti-DENV NS1 HuMAbs induce ZIKV neutralization in endothelial cells: HUVECs are infected with ZIKV (MOI = 0.125) at 24 and 48 h post-infection. To determine the neutralization potency represented by the percentage of viral reduction in Vero cells, culture supernatants from four HuMAbs against DENV NS1 (**A**) M20; (**B**) M1; (**C**) 238 and (**D**) 8-treated infected cells are collected. The results are presented as the mean ± SD of duplicate independent experiments.

To clarify whether DENV NS1 HuMAbs neutralized infected cells, HMEC-1 and HUVECs were infected with DENV serotypes 1–4 or ZIKV, respectively, and treated with various concentrations of HuMAbs. The percentage of viral reduction in Vero cells determined the neutralizing ability of the antibodies. The results showed that viral clearance appeared at 24 h post-infection, which was dose-dependent (Figure 1A–M). For DENV 1–3 at 24 h post-infection, the inhibitory concentrations (IC_50_) were 26.36–55.19, 35.37–82.55, 31.70–86.33, and 5.23–47.17 µg/mL after treatment with HuMAb M20 (Figure 1A,E,I), HuMAb M1 (Figure 1B,F,J), HuMAb 238 (Figure 1C,G,K), and HuMAb 8 (Figure 1M), respectively. Increasing neutralization was observed at 48 h post-infection with IC_50_ values of 20.93–61.39, 32.96–83.60, 32.07–82.78, and 7.40–25.60 µg/mL with HuMAb M20, M1, 238, and 8, respectively. However, HuMAb 8 was cross-reactive with DENV serotype 4 with IC_50_ of 5.02 and 4.82 µg/mL at 24 and 48 h post-infection (Figure 1M). Notably, HuMAb 8 bound specifically to DENV NS1 and E-protein (Figure S1) and had a highly effective neutralization effect against all dengue serotypes, particularly DENV 2 and DENV 4. In contrast, ZIKV was resistant to all four HuMAbs compared with DENV infection (Figure 2A–D); however, HuMAb 8 still provided good neutralization, with IC_50_ of 20.21 and 83.99 µg/mL at 24 and 48 h post-infection. These results suggest that the four DENV NS1 HuMAbs, particularly HuMAb 8, had a better inhibitory effect against DENV 1–4 infection than ZIKV.

### 3.2. DENV NS1 HuMAbs Stimulate Complement-Mediated Cell Cytolysis and Inhibit the Viral Replication of Infected Cells

**Figure 3:**
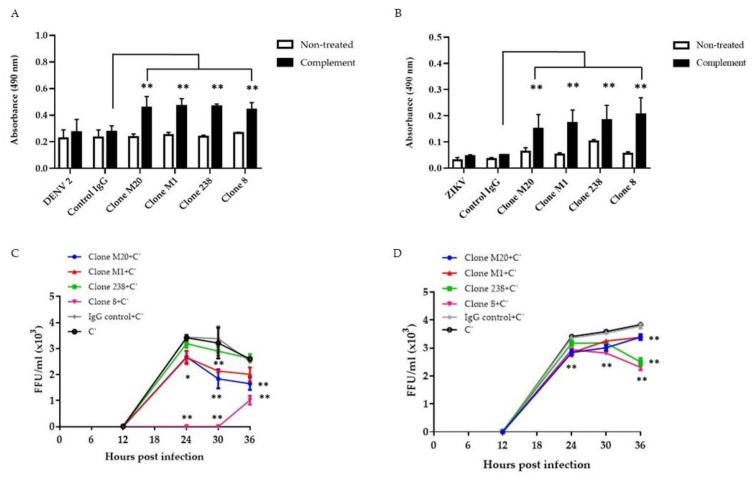
Anti-DENV NS1 HuMAbs stimulate complement-mediated cell cytolysis and inhibition of viral replication: HMEC-1 and HUVECs are infected with (**A**) DENV serotype 2 and (**B**) ZIKV (MOI = 5.0), respectively. At 36 h post-infection, cells are incubated with four anti-DENV NS1 HuMAbs (clones M20, M1, 238, and 8) (100 µg/mL) or control IgG for 30 min at 37 °C with complement (C’) for 4 h at 37 °C. The culture supernatants from all groups under each condition are determined for cell cytolysis by detecting the release of lactate dehydrogenase (LDH) (** *p*-value < 0.01). At 6 h post-infection, (**C**) DENV-infected HMEC-1 or (**D**) ZIKV-infected HUVECs are incubated with four anti-DENV NS1 HuMAbs (M20, M1, 238, and 8) (100 µg/mL) or control IgG at different timepoints with complement addition. Viral titers in supernatants are measured using focus forming assay as FFU/mL. The results presented as the mean ± SD of duplicate independent experiments in different time points (* *p*-value < 0.05; ** *p*-value < 0.01 compared with C’ plus control IgG).

Several studies have revealed that anti-NS1 Abs remove infected cells via the complement pathway [18,21,22,23]. Binding between Abs and NS1 on the surface of DENV-infected cells enhanced complement activation and cytolysis [18]. Herein, we confirmed the potential effects of DENV NS1 HuMAbs on flavivirus infection in different endothelial cells. The complement had no significant effect on infected cells or control IgG treated cells (Figure 3A,B). Compared with control IgG administration, the four DENV NS1 HuMAbs significantly enhanced complement-mediated cell cytolysis in DENV-infected HMEC-1 (Figure 3A) and ZIKV-infected HUVECs (Figure 3B) as measured by LDH enzyme release. Wan et al. demonstrated that anti-DENV NS1 Abs cooperated with complement to reduce viral output from the infected cells [18]. To clarify whether these DENV NS1 HuMAbs affected viral replication via complement, DENV-infected HMEC-1 and ZIKV-infected HUVECs were treated with HuMAbs plus complement. At the indicated time points, viral release into the culture medium was measured by FFU assays. At 30 h post-infection of DENV and ZIKV, DENV NS1 HuMAbs displayed significant viral diminution compared with control IgG treated with complement or complement alone (Figure 3C,D). Of note, complete inhibition was observed when DENV-infected cells were treated with HuMAb 8 combined with complement (Figure 3C). These viral titers were recovered 30 h post-infection. At 36 h post-infection, compared with HuMAb M1 and 238, HuMAb M20 and 8 combined with complement provided the greatest viral reduction (Figure 3C). However, HuMAb 238 and 8 plus complement treatment of ZIKV-infected cells had a greater effect on reducing viral release than HuMAb M20 and M1 treatment at 36 h post-infection (Figure 3D). These results indicate that anti-DENV NS1 HuMAbs stimulate the complement-mediated cell cytolysis of infected cells that present the NS1 protein on their surface, leading to the disturbance of DENV and ZIKV replication.

### 3.3. Anti-DENV NS1 HuMAbs Protect against NS1-Induced Endothelial Leakage

**Figure 4:**
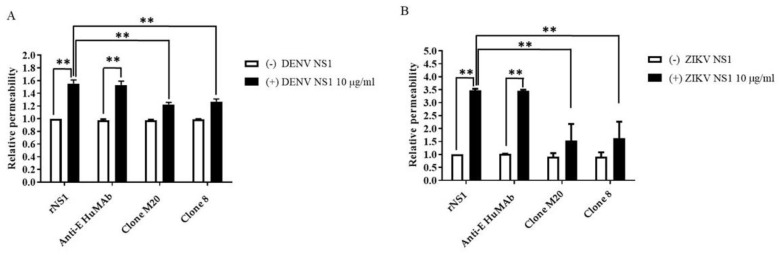
DENV NS1 HuMAbs protect NS1-mediated endothelial leakage in vitro: monolayer of HMEC-1 and HUVECs cells are grown on Transwell inserts and incubated with (**A**) DENV NS1 or (**B**) ZIKV NS1 alone or NS1 with either anti-E HuMAb or anti-DENV NS1 HuMAbs; M20 and 8. Untreated cells are used as a negative control. After 24 h treatment, endothelial permeability has been evaluated by measuring the HRP activity. The results are presented as the average of mean ± SD of two independent experiments. ** *p*-value < 0.01 indicates a significant difference.

NS1 is the viral toxin counterpart of bacterial endotoxin lipopolysaccharide, which contributes to vascular leakage in patients with dengue [13]. A previous study reported that NS1-immune serum and anti-NS1 mouse Abs blocked NS1-induced endothelial permeability [15]. To test the potential effects of DENV NS1 HuMAbs against NS1-triggered endothelial dysfunction, DENV NS1 and ZIKV NS1 alone (10 µg/mL) were incubated with HMEC-1 and HUVECs, respectively. Both cell lines were cultured on a Transwell permeable membrane as a supportive device to imitate cell barrier functions in vitro. Endothelial changes were examined by measuring the HRP activity. Untreated cells were used as a negative control. The incubation of NS1 (10 µg/mL) markedly disrupted the integrity of HMEC-1 (Figure 4A) and HUVECs (Figure 4B). Expectedly, anti-E HuMAb did not inhibit NS1-induced cell permeability. Notably, DENV NS1 HuMAb M20 and 8 significantly blocked the ability of NS1 to trigger endothelial leakage compared with no treatment or NS1 administration alone (Figure 4A). Similar results were observed in HUVECs incubated with ZIKV NS1 (Figure 4B), indicating that DENV NS1 HuMAbs have a potential therapeutic effect against flavivirus NS1-induced endothelial permeability.

### 3.4. Effect of DENV NS1 HuMAbs on NS1-Mediated Inflammatory Cytokine Secretion In Vitro

**Figure 5:**
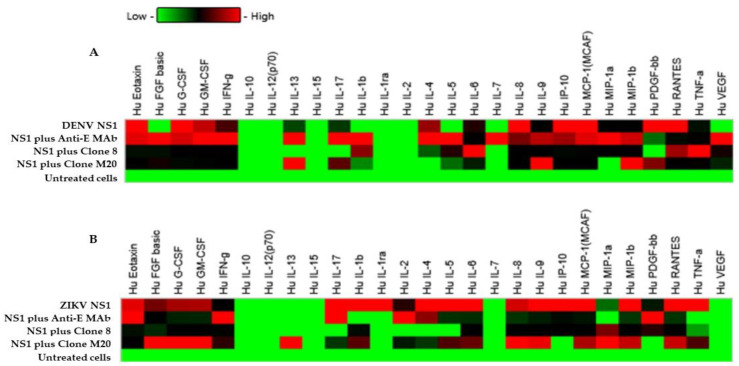
Effect of DENV NS1 HuMAbs to NS1-mediated inflammatory cytokines secretion in vitro: HMEC-1 and HUVECs cells were grown on Transwell inserts and incubated with (**A**) DENV NS1 and (**B**) ZIKV NS1 alone or NS1 with either anti-E HuMAb or anti-DENV NS1 HuMAbs; M20 and 8. Untreated cells are used as a negative control. The heatmap of cytokine production of DENV NS1-treated HMEC-1 cells and ZIKV NS1-treated HUVECs cells are analyzed, and the level of cytokine series is measured using human cytokine multiplex assay at 24 h post-treatment. Data are derived from two independent experiments, each with duplicate samples.

Cytokine storm is a pathogenic mechanism that causes severe dengue disease [24]. During DENV infection, the augmentation of several proinflammatory cytokines may contribute to vascular permeability through glycocalyx dysfunction and the tight junction disruption of [25,26,27]. The endothelial glycocalyx layer (EGL) has a key role in regulating the function of the endothelial barrier, which contains a carbohydrate-rich layer that lines the vascular endothelium. DENV NS1 induced EGL dysfunction leading to the secretion of proinflammatory cytokines and vascular leakage [28]. To determine whether cytokines were associated with flavivirus NS1-mediated endothelial hyperpermeability in vitro, we stimulated HMEC-1 and HUVECs with DENV NS1 and ZIKV NS1 (10 µg/mL), respectively, and subsequently treated them with DENV NS1 HuMAb M20 and 8. Our experiments mimicked the pathological conditions in DENV-infected patients in whom the estimated range of circulating NS1 in the serum is 0.01 to 50 μg/mL [29]. Cell cultures were collected at 24 h post-treatment and untreated cells were used as a negative control. After cells were activated with the NS1 protein, the secretion of most cytokines showed an increasing trend. Among 27 cytokines, 21 and 22 cytokines were expressed in DENV NS1-treated HMEC-1 (Figure 5A) and ZIKV NS1-treated HUVECs (Figure 5B), respectively, under all conditions. IL-1ra, IL-2, IL-7, IL-10, IL-12, IL-15, and VEGF were not detected, whereas IL-8 showed dominant secretion from both cell lines, which demonstrated significant difference compared to untreated cells (Table 3). Interestingly, DENV NS1 HuMAbs abolished several cytokines from HMEC-1 (Figure 5A). In contrast, the secretion of 13 cytokines (IL-1b, IL-1ra, IL-2, IL-4, IL-5, IL-6, IL-9, IL-17, eotaxin, IP-10, MCP-1, MIP-1β, and TNF-α) was decreased in ZIKV NS1-treated HUVECs (Figure 5B). However, a reduction in most cytokines was observed following treatment with anti-E MAb. This suggests that DENV NS1 and ZIKV NS1 administration in different endothelial cells provides similar and dissimilar patterns of cytokine production, which may be related to endothelial leakage (Figure 4). A similar reduction in NS1-induced secretion was elicited for three cytokines (eotaxin, IP-10, and MCP-1) in different endothelial cells. Notably, treatment with HuMAbs, particularly HuMAb8, induced obvious changes in the decreases of IP-10 secretion.

### 3.5. Mapping of NS1-Specific HuMAbs

**Figure 6:**
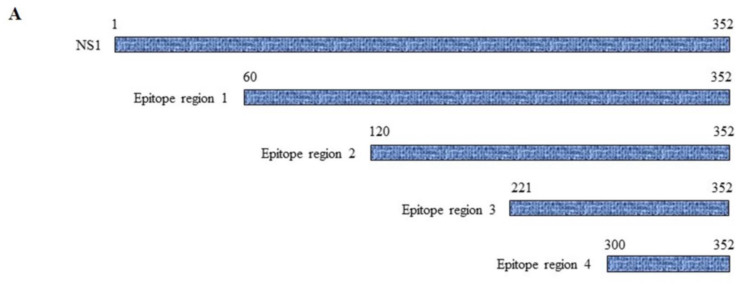

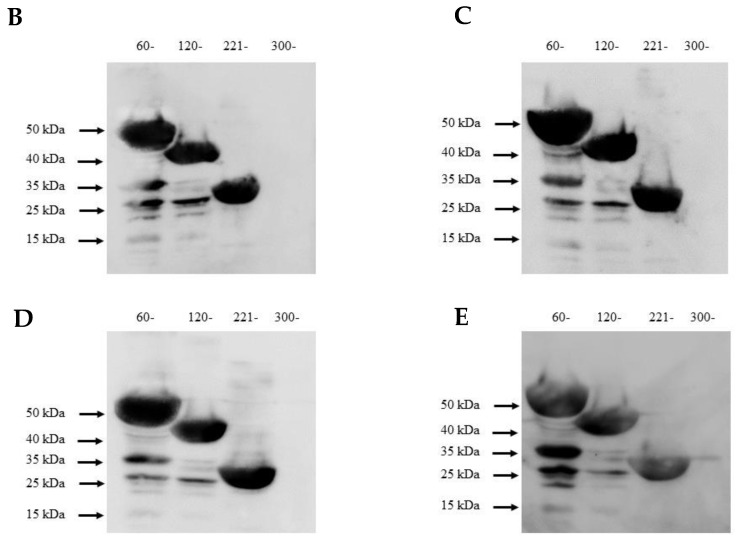
Epitope mapping of anti-DENV NS1 HuMAbs: (**A**) The truncated NS1 protein regions are constructed and expressed in *E. Coli* BL21DE3. Lysates expressing truncated NS1 proteins are loaded to SDS-PAGE, and the proteins were transferred to the PVDF membrane. Each epitope regions is subsequently stained with DENV NS1 HuMAbs (**B**) HuMAb M20; (**C**) HuMAb M1; (**D**) HuMAb 238, and (**E**) HuMAb 8.

Because molecular mimicry between DENV NS1 protein and human antigens was proposed to mediate the DENV pathogenesis [30,31,32] and cross-reactive antibodies were predominantly noted at the C-terminal region of DENV NS1 [33,34], we examined the epitope regions recognized by our four DENV NS1 HuMAbs using a series of truncated DENV2 NS1 proteins (Figure 6A). Each construct contained a region of NS1 at 60–352, 120–352, 221–352, and 300–352 amino acids. The four HuMAbs reacted with all the truncated NS1 proteins except for one region (300–352) (Figure 6B–E), indicating that these HuMAbs recognize a C-terminal region at 221–299 amino acids in the DENV 2 NS1 protein.

## 4. Discussion

As epidemic and seasonal outbreaks of flaviviruses have increased, several studies have highlighted the generation of neutralizing antibodies against the envelope protein of flaviviruses for therapeutic or vaccine strategies [35,36]. However, anti-envelope antibodies are related to the ADE phenomenon [37,38]. Antibodies against NS1 that lack the risk of ADE have been developed as an alternative therapy for dengue [22,39,40,41,42]. However, NS1-mediated endothelial dysfunction was reported for flaviviruses [43,44], and some anti-NS1 Abs may facilitate dengue pathogenesis through different mechanisms by cross-reacting with self-antigens [31,32,45,46,47,48,49] to disturb the intracellular tight junctions of endothelial cells. Thus, the development and characterization of NS1-specific antibodies for dengue treatment should be thoroughly studied.

In this study, our DENV NS1 HuMAbs exhibited viral neutralization at various time points, particularly HuMAb clone 8, which extensively reduced the viral load of all dengue serotypes without complement addition. In contrast, all antibodies had low viral neutralization for the ZIKV infection, showing that all antibodies tested were more specific for DENV NS1 than for ZIKV. However, all three HuMAbs (M20, M1, and 238) with complement showed similar protective effects. Anti-NS1 HuMAb clone 8 provided the greatest inhibition of DENV replication at 30 h post-infection, and viral titers were recovered at 36 h post-infection, which was different from the other antibodies, which reduced viral replication with extended incubation times. This phenomenon may be related to the cross-reactivity of anti-NS1 HuMAb clone 8 to the E-protein of DENV resulting in the enhanced ability of viral proliferation through the Fcγ receptor. However, our study demonstrates that anti-DENV NS1 HuMAbs derived from humans can mediate complement-dependent cytolysis and control the viral load. Consistent with the result of previous studies, anti-NS1 Abs and depleted C-terminal NS1 Abs reduced local skin hemorrhage, controlled the viral load of DENV infection, and inhibited viral replication and cytotoxicity via complement [18,19]. Moreover, NS1 antibodies triggered phagocytosis and viral clearance through the Fcγ receptor in patients infected with West Nile virus [40], indicating that NS1 is essential for viral entry, replication, assembly, release, and is therefore related to viral morphogenesis [8,50,51].

A noteworthy finding in this study is that DENV NS1 HuMAb M20 and 8 decreased NS1-associated vascular leakage. By extension, NS1 obviously reversed the strength of the endothelial barrier. This is consistent with the result of previous studies wherein NS1 mediated endothelial dysfunction [15,28,43]; however, our antibodies did not trigger endothelial permeability without the presence of the NS1 protein. Interestingly, compared with anti-E antibody treatment, DENV NS1 HuMAb M20 and 8 significantly protected the endothelial cells from DENV and ZIKV NS1-induced endothelial permeability. Regarding NS1-induced pathogenesis, NS1 may also stimulate inflammatory cytokine production leading to vascular leakage. Supporting studies demonstrated that NS1 activated target cells through TLR4 [14] to induce endothelial disruption [16,52], as well as TLR2/TLR6 [53], resulting in cytokine production. Moreover, several studies have reported the ability of NS1 to induce IL-6, IL-8, TNF-α, MCP-1, RANTES, and IP-10 in vitro and in vivo [14,15,16,54,55]. Chemokines (MIP-1, RANTES, and IP-10) were augmented in the plasma of patients with dengue [56]. Additionally, the high MCP-1 expression was observed in patients with severe dengue and was believed to promote immune cell infiltration and migration. This may affect endothelial cells, leading to vascular leakage [27,55,57,58]. Herein, we have observed that seven cytokines (IL-1ra, IL-2, IL-7, IL-10, IL-12, IL-15, and VEGF) were not affected by the NS1 protein or NS1 plus antibody treatment. DENV NS1 HuMAb M20 and 8 decreased the secretion of NS1-induced cytokines, particularly IL-8, IP-10, MCP-1, and RANTES, from endothelial cells. Typically, IL-8 and MCP-1 were detected at high levels in patients with severe dengue. These chemokines manipulate transmembrane permeability by regulating the tight junctions and cytoskeletons of endothelial cells. IL-8 is a significant vasoactive mediator that regulates the activation of adhesion molecules in endothelial cells [59,60]. Nevertheless, the pretreatment of DENV-infected monocytes with IL-8 and MCP-1 antibodies showed a partial reversal in endothelial integrity indicating the effect of chemokines in this process [61,62]. In contrast with a study by Glasner et al., HMEC-1 did not produce IL-6, TNF-α, or IL-8 in response to DENV2 NS1 compared with untreated cells. Furthermore, DENV2 NS1 triggered similar levels of vascular leakage in TNF-αR-deficient B6 mice and wild-type mice, suggesting that endothelial permeability was independent of inflammatory cytokines [63]. However, they also observed that recombinant IL-6 and TNF-α induced endothelial leakage, which was reduced after blocking the production of these cytokines. Therefore, inflammatory mediator-related pathogenesis may promote paracrine and autocrine synergistic effects inside cells [64,65]. Further research is required to fully elucidate whether cytokines drive NS1-related pathogenesis.

Flavivirus NS1 consists of three domains; however, which of these are responsible for distinct pathogenic or therapeutic functions remains unknown. Therefore, the rational design of antibody-specific NS1 has been investigated. Active immunization with NS1 protein and NS1 antibodies from passive immunization revealed a protective effect against DENV in mice [15,18,41,66,67,68]. A study of 2B7 (a mouse monoclonal antibody against DENV NS1 immunoglobulin G2b) reported that the NS1 wing domain, particularly the WWG motif (amino acid residues 115, 118, and 119), was essential for initial cell attachment, whereas the tip of the β-ladder (amino acid residues 301, 303, 305, and 326–327) was critical for the downstream process of NS1-mediated endothelial dysfunction [68]. Several studies revealed that cross-reactive antibodies recognized the C-terminal region of DENV NS1 [33,34]. Our study demonstrated that all four anti-DENV NS1 HuMAbs (M20, M1, 238, and 8) recognized the C-terminal site (221–299 amino acids) at the β-ladder domain, which had a protective effect against the pathogenesis of DENV and ZIKV infections. Currently, the precise mechanism underlying the inhibition of DENV and ZIKV infections remain unknown.

To eliminate the potential risk of ADE and prevent viral infection, anti-NS1 HuMAb can be used alone or in combination with the modified Fc of anti-E MAb (cocktail MAbs). DENV NS1 HuMAb M20 stimulated the cytolysis of infected endothelial cells (HMEC-1 and HUVECs) in the presence of the complement and inhibited vascular permeability, suggesting that HuMAbs from the full-length DENV NS1 do not mediate dengue pathogenesis in vitro. Nevertheless, DENV NS1 HuMAb 8 showed not only the reduction of endothelial leakage, which is the distinguished NS1 pathogenesis, but also the enhancement of viral neutralization as a result of its cross-reactivity to the E-protein, suggesting that the Fc portion should be eliminated.

## 5. Conclusions

**Figure 7:**
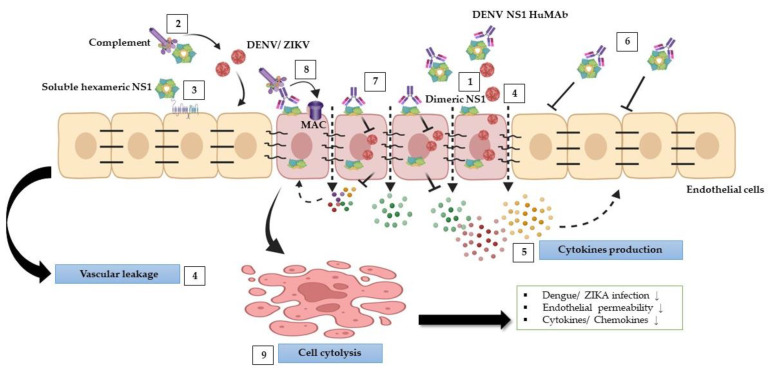
Schematic model of protective effects stimulated by DENV NS1 HuMAbs: during DENV and ZIKV infections, NS1 mediates multiple pathogenic pathways. (1) NS1 plays a role in DENV and ZIKV replication within cells. (2) NS1 disturbs complement proteins to bind to viruses leading viruses to escape from the complement attack. (3) Soluble NS1 interaction with endothelial cells leads to increased glycocalyx degradation. (4) Furthermore, NS1 modulates intercellular junction molecules, resulting in endothelial dysfunction and vascular leakage. (5) NS1 can activate inflammation by triggering several proinflammatory cytokines leading to cytokine storm, which may enhance DENV and ZIKV infections and vascular leakage. (6) Anti-DENV NS1 HuMAb directly binds to soluble NS1 and supports viral neutralization. (7) The binding of DENV NS1 HuMAb to dimeric NS1 on the infected cell surface inhibits DENV and ZIKV replication. (8) NS1 and anti-DENV NS1 HuMAb interaction with the infected cells can stimulate complement membrane attack complex (MAC) formation and mediate cell cytolysis (9) that may attenuate DENV and ZIKV infections, endothelial permeability, and cytokines/chemokines secretion.

Our findings indicate that anti-DENV NS1 HuMAbs reduced flavivirus-triggered pathological effects (Figure 7), in part by neutralizing the virus and enhancing cell cytolysis via complement pathway to activate the membrane attack complex, resulting in a decrease in viral replication, endothelial permeability, and cytokine/chemokine secretion. This study suggests that HuMAbs against DENV NS1 may have broad therapeutic benefits for the prevention of severe dengue and Zika infections.

## Figures and Tables

**Table 1:** Summary of patients’s backgrounds and HuMAbs used in this study.

Patients	Gender	Age	Diagnosis	Blood Collection		Rapid Test		PCR Serotyping	HuMAbs
				Days	Phase	IgG	IgM		
D25	Male	27	DF	14	Convalescent	+	+	DENV2	8, M1, M20
D26	Female	33	DF	19	Convalescent	+	+	DENV2	238

**Table 2:** Primers for the construction of cDNA clones.

Primer	Sequence
DVNS1 60-Fw	5-GGC GGA TCC ATG GTA ACA AGA CTG GAA AAT CTG-3
DVNS1 120-Fw	5-GGC GGA TCC ATG AAA GCG AAA ATG CTC TCT ACA GAG-3
DVNS1 221-Fw	5-GGC GGA TCC ATG AAA AGC TGC CAC TGG CCA AAG-3
DVNS1 300-Fw	5-GGC GGA TCC ATG ACA ACT ACT GCC TCT GGA AAA CTC-3
DVNS1 352-Rv	5-CCG CTC GAG TTA GGC TGT GAC CAA GGA GTT GAC-3

**Table 3:** Statistical analysis of effect of DENV NS1 HuMAbs to both DENV NS1 and ZIKV NS1-mediated inflammatory cytokines secretion in vitro compared to untreated cells.

Cytokines	NS1		NS1 Plus Anti-E mAb		NS1 Plus DENV NS1 HuMAb-Clone M20		NS1 Plus DENV NS1 HuMAb-Clone 8	
	DENV	ZIKV	DENV	ZIKV	DENV	ZIKV	DENV	ZIKV
IL-1β	NS	NS	NS	NS	NS	NS	NS	NS
IL-1ra	NS	NS	NS	NS	NS	NS	NS	NS
IL-2	NS	NS	NS	NS	NS	NS	NS	NS
IL-4	NS	NS	NS	NS	NS	NS	NS	NS
IL-5	NS	NS	NS	NS	NS	NS	NS	NS
IL-6	NS	NS	NS	NS	NS	NS	NS	NS
IL-7	NS	NS	NS	NS	NS	NS	NS	NS
IL-8	<0.01	<0.01	<0.01	<0.01	<0.01	<0.01	<0.01	<0.01
IL-9	NS	NS	NS	NS	NS	NS	NS	NS
IL-10	NS	NS	NS	NS	NS	NS	NS	NS
IL-12	NS	NS	NS	NS	NS	NS	NS	NS
IL-13	NS	NS	NS	NS	NS	NS	NS	NS
IL-15	NS	NS	NS	NS	NS	NS	NS	NS
IL-17	NS	NS	NS	NS	NS	NS	NS	NS
Eotaxin	NS	NS	NS	NS	NS	NS	NS	NS
FGF-β	NS	NS	NS	NS	NS	NS	NS	NS
G-CSF	NS	NS	NS	NS	NS	NS	NS	NS
GM-CSF	NS	NS	NS	NS	NS	NS	NS	NS
IFN-γ	NS	NS	NS	NS	NS	NS	NS	NS
IP-10	<0.01	<0.01	<0.01	NS	<0.05	<0.05	<0.05	<0.05
MCP-1	<0.01	<0.05	<0.01	NS	<0.01	<0.05	<0.01	NS
MIP-1α	NS	NS	NS	NS	NS	NS	NS	NS
PDGF	NS	NS	NS	NS	NS	NS	NS	NS
MIP-1β	NS	NS	NS	NS	NS	NS	NS	NS
RANTES	NS	NS	NS	NS	NS	NS	NS	NS
TNF-α	NS	NS	NS	NS	NS	NS	NS	NS
VEGF	NS	NS	NS	NS	NS	NS	NS	NS

## Data Availability

The data presented in this study are available on request from the corresponding author.

## Supplementary Materials

The following supporting information can be downloaded at: https://www.mdpi.com/article/10.3390/biomedicines11010227/s1, Figure S1. Recognition of native NS1 protein from DENV2 by HuMabs using western blot.

## References

[1] Kyle J.L., Harris E. (2008). Global Spread and Persistence of Dengue. Annu. Rev. Microbiol..

[2] Gould E.A., Solomon T. (2008). Pathogenic Flaviviruses. Lancet.

[3] Counotte M.J., Egli-Gany D., Riesen M., Abraha M., Porgo T.V., Wang J., Low N. (2018). Zika Virus Infection as a Cause of Congenital Brain Abnormalities and Guillain-Barre Syndrome: From Systematic Review to Living Systematic Review. F1000Research.

[4] Rodriguez-Barraquer I., Costa F., Nascimento E.J.M., Nery N.J., Castanha P.M.S., Sacramento G.A., Cruz J., Carvalho M., De Olivera D., Hagan J.E. (2019). Impact of Preexisting Dengue Immunity on Zika Virus Emergence in a Dengue Endemic Region. Science.

[5] Kuhn R.J., Zhang W., Rossmann M.G., Pletnev S.V., Corver J., Lenches E., Jones C.T., Mukhopadhyay S., Chipman P.R., Strauss E.G. (2002). Structure of Dengue Virus: Implications for Flavivirus Organization, Maturation, and Fusion. Cell.

[6] World Health Organization (2017). Dengue Vaccine: WHO Position Paper, July 2016—Recommendations. Vaccine.

[7] Avirutnan P., Zhang L., Punyadee N., Manuyakorn A., Puttikhunt C., Kasinrerk W., Malasit P., Atkinson J.P., Diamond M.S. (2007). Secreted NS1 of Dengue Virus Attaches to the Surface of Cells via Interactions with Heparan Sulfate and Chondroitin Sulfate E. PLoS Pathog..

[8] Muller D.A., Young P.R. (2013). The Flavivirus NS1 Protein: Molecular and Structural Biology, Immunology, Role in Pathogenesis and Application as a Diagnostic Biomarker. Antivir. Res..

[9] Song H., Qi J., Haywood J., Shi Y., Gao G.F. (2016). Zika Virus NS1 Structure Reveals Diversity of Electrostatic Surfaces Among Flaviviruses. Nat. Struct. Mol. Biol..

[10] Mackenzie J.M., Jones M.K., Young P.R. (1996). Immunolocalization of the Dengue Virus Nonstructural Glycoprotein NS1 Suggests a Role in Viral RNA Replication. Virology.

[11] Avirutnan P., Fuchs A., Hauhart R.E., Somnuke P., Youn S., Diamond M.S., Atkinson J.P. (2010). Antagonism of the Complement Component C4 by Flavivirus Nonstructural Protein NS1. J. Exp. Med..

[12] Chuang Y.C., Wang S.Y., Lin Y.S., Chen H.R., Yeh T.M. (2013). Re-Evaluation of the Pathogenic Roles of Nonstructural Protein 1 and Its Antibodies During Dengue Virus Infection. J. Biomed. Sci..

[13] Modhiran N., Watterson D., Muller D.A., Panetta A.K., Sester D.P., Liu L., Hume D.A., Stacey K.J., Young P.R. (2015). Dengue Virus NS1 Protein Activates Cells via Toll-Like Receptor 4 and Disrupts Endothelial Cell Monolayer Integrity. Sci. Transl. Med..

[14] Modhiran N., Watterson D., Blumenthal A., Baxter A.G., Young P.R., Stacey K.J. (2017). Dengue Virus NS1 Protein Activates Immune Cells via TLR4 but Not TLR2 or TLR6. Immunol. Cell Biol..

[15] Beatty P.R., Puerta-Guardo H., Killingbeck S.S., Glasner D.R., Hopkins K., Harris E. (2015). Dengue Virus NS1 Triggers Endothelial Permeability and Vascular Leak That Is Prevented by NS1 Vaccination. Sci. Transl. Med..

[16] Chen H.R., Chuang Y.C., Lin Y.S., Liu H.S., Liu C.C., Perng G.C., Yeh T.M. (2016). Dengue Virus Nonstructural Protein 1 Induces Vascular Leakage Through Macrophage Migration Inhibitory Factor and Autophagy. PLoS Negl. Trop. Dis..

[17] Chen H.R., Lai Y.C., Yeh T.M. (2018). Dengue Virus Non-structural Protein 1: A Pathogenic Factor, Therapeutic Target, and Vaccine Candidate. J. Biomed. Sci..

[18] Wan S.W., Lu Y.T., Huang C.H., Lin C.F., Anderson R., Liu H.S., Yeh T.M., Yen Y.T., Wu-Hsieh B.A., Lin Y.S. (2014). Protection Against Dengue Virus Infection in Mice by Administration of Antibodies Against Modified Nonstructural Protein 1. PLoS ONE.

[19] Wan S.W., Chen P.W., Chen C.Y., Lai Y.C., Chu Y.T., Hung C.Y., Lee H., Wu H.F., Chuang Y.C., Lin J. (2017). Therapeutic Effects of Monoclonal Antibody Against Dengue Virus NS1 in a STAT1 Knockout Mouse Model of Dengue Infection. J. Immunol..

[20] Setthapramote C., Sasaki T., Puiprom O., Limkittikul K., Pitaksajjakul P., Pipattanaboon C., Sasayama M., Leuangwutiwong P., Phumratanaprapin W., Chamnachanan S. (2012). Human Monoclonal Antibodies to Neutralize All Dengue Virus Serotypes Using Lymphocytes from Patients at Acute Phase of the Secondary Infection. Biochem. Biophys. Res. Commun..

[21] Schlesinger J.J., Brandriss M.W., Walsh E.E. (1985). Protection Against 17-D Yellow Fever Encephalitis in Mice by Passive Transfer of Monoclonal Antibodies to the Nonstructural Glycoprotein gp48 and by Active Immunization with gp48. J. Immunol..

[22] Lin Y.L., Chen L.K., Liao C.L., Yeh C.T., Ma S.H., Chen J.L., Huang Y.L., Chen S.S., Chiang H.Y. (1998). DNA Immunization with Japanese Encephalitis Virus Nonstructural Protein NS1 Elicits Protective Immunity in Mice. J. Virol..

[23] Kitai Y., Kondo T., Konishi E. (2010). Complement-Dependent Cytotoxicity Assay for Differentiating West Nile Virus from Japanese Encephalitis Virus Infections in Horses. Clin. Vaccine Immunol..

[24] Rothman A.L. (2011). Immunity to Dengue Virus: A Tale of Original Antigenic sin and Tropical Cytokine Storms. Nat. Rev. Immunol..

[25] Appanna R., Wang S.M., Ponnampalavanar S.A., Lum L.C., Sekaran S.D. (2012). Cytokine Factors Present in Dengue Patient Sera Induces Alterations of Junctional Proteins in Human Endothelial Cells. Am. J. Trop. Med. Hyg..

[26] Dewi B.E., Takasaki T., Kurane I. (2004). In Vitro Assessment of Human Endothelial Cell Permeability: Effects of Inflammatory Cytokines and Dengue Virus Infection. J. Virol. Methods.

[27] Lee Y.R., Liu M.T., Lei H.Y., Liu C.C., Wu J.M., Tung Y.C., Lin Y.S., Yeh T.M., Chen S.H., Liu H.S. (2006). MCP-1, a Highly Expressed Chemokine in Dengue Haemorrhagic Fever/Dengue Shock Syndrome Patients, May Cause Permeability Change, Possibly Through Reduced Tight Junctions of Vascular Endothelium Cells. J. Gen. Virol..

[28] Puerta-Guardo H., Glasner D.R., Harris E. (2016). Dengue Virus NS1 Disrupts the Endothelial Glycocalyx, Leading to Hyperpermeability. PLoS Pathog..

[29] Alcon S., Talarmin A., Debruyne M., Falconar A., Deubel V., Flamand M. (2002). Enzyme-Linked Immunosorbent Assay Specific to Dengue Virus Type 1 Nonstructural Protein NS1 Reveals Circulation of the Antigen in the Blood During the Acute Phase of Disease in Patients Experiencing Primary or Secondary Infections. J. Clin. Microbiol..

[30] Lin C.F., Lei H.Y., Shiau A.L., Liu H.S., Yeh T.M., Chen S.H., Liu C.C., Chiu S.C., Lin Y.S. (2002). Endothelial Cell Apoptosis Induced by Antibodies Against Dengue Virus Nonstructural Protein 1 via Production of Nitric Oxide. J. Immunol..

[31] Lin C.F., Lei H.Y., Shiau A.L., Liu C.C., Liu H.S., Yeh T.M., Chen S.H., Lin Y.S. (2003). Antibodies from Dengue Patient Sera Cross-React with Endothelial Cells and Induce Damage. J. Med. Virol..

[32] Lin Y.S., Yeh T.M., Lin C.F., Wan S.W., Chuang Y.C., Hsu T.K., Liu H.S., Liu C.C., Anderson R., Lei H.Y. (2011). Molecular Mimicry Between Virus and Host and Its Implications for Dengue Disease Pathogenesis. Exp. Biol. Med. (Maywood).

[33] Cheng H.J., Lin C.F., Lei H.Y., Liu H.S., Yeh T.M., Luo Y.H., Lin Y.S. (2009). Proteomic Analysis of Endothelial Cell Autoantigens Recognized by Anti-dengue Virus Nonstructural Protein 1 Antibodies. Exp. Biol. Med. (Maywood).

[34] Chen M.C., Lin C.F., Lei H.Y., Lin S.C., Liu H.S., Yeh T.M., Anderson R., Lin Y.S. (2009). Deletion of the C-Terminal Region of Dengue Virus Nonstructural Protein 1 (NS1) Abolishes Anti-NS1-Mediated Platelet Dysfunction and Bleeding Tendency. J. Immunol..

[35] De Alwis R., Smith S.A., Olivarez N.P., Messer W.B., Huynh J.P., Wahala W.M., White L.J., Diamond M.S., Baric R.S., Crowe J.E. (2012). Identification of Human Neutralizing Antibodies That Bind to Complex Epitopes on Dengue Virions. Proc. Natl. Acad. Sci. USA.

[36] Barba-Spaeth G., Dejnirattisai W., Rouvinski A., Vaney M.C., Medits I., Sharma A., Simon-Lorière E., Sakuntabhai A., Cao-Lormeau V.M., Haouz A. (2016). Structural Basis of Potent Zika-Dengue Virus Antibody Cross-Neutralization. Nature.

[37] Halstead S.B. (2003). Neutralization and Antibody-Dependent Enhancement of Dengue Viruses. Adv. Virus Res..

[38] Sridhar S., Luedtke A., Langevin E., Zhu M., Bonaparte M., Machabert T., Savarino S., Zambrano B., Moureau A., Khromava A. (2018). Effect of Dengue Serostatus on Dengue Vaccine Safety and Efficacy. N. Engl. J. Med..

[39] Schlesinger J.J., Foltzer M., Chapman S. (1993). The Fc Portion of Antibody to Yellow Fever Virus NS1 Is a Determinant of Protection Against YF Encephalitis in Mice. Virology.

[40] Chung K.M., Nybakken G.E., Thompson B.S., Engle M.J., Marri A., Fremont D.H., Diamond M.S. (2006). Antibodies Against West Nile Virus Nonstructural Protein NS1 Prevent Lethal Infection Through Fc Gamma Receptor-Dependent and -Independent Mechanisms. J. Virol..

[41] Henchal E.A., Henchal L.S., Schlesinger J.J. (1988). Synergistic Interactions of Anti-NS1 Monoclonal Antibodies Protect Passively Immunized Mice from Lethal Challenge with Dengue 2 Virus. J. Gen. Virol..

[42] Falgout B., Bray M., Schlesinger J.J., Lai C.J. (1990). Immunization of Mice with Recombinant Vaccinia Virus Expressing Authentic Dengue Virus Nonstructural Protein NS1 Protects Against Lethal Dengue Virus Encephalitis. J. Virol..

[43] Puerta-Guardo H., Glasner D.R., Espinosa D.A., Biering S.B., Patana M., Ratnasiri K., Wang C., Beatty P.R., Harris E. (2019). Flavivirus NS1 Triggers Tissue-Specific Vascular Endothelial Dysfunction Reflecting Disease Tropism. Cell Rep..

[44] Puerta-Guardo H., Tabata T., Petitt M., Dimitrova M., Glasner D.R., Pereira L., Harris E., Virus Z. (2020). Zika Virus Nonstructural Protein 1 Disrupts Glycosaminoglycans and Causes Permeability in Developing Human Placentas. J. Infect. Dis..

[45] Falconar A.K. (1997). The Dengue Virus nonstructural-1 Protein (NS1) Generates Antibodies to Common Epitopes on Human Blood Clotting, Integrin/Adhesin Proteins and Binds to Human Endothelial Cells: Potential Implications in Haemorrhagic Fever Pathogenesis. Arch. Virol..

[46] Huang K.J., Li S.J., Chen S.C., Liu H.S., Lin Y.S., Yeh T.M., Liu C.C., Lei H.Y. (2000). Manifestation of Thrombocytopenia in dengue-2-virus-infected Mice. J. Gen. Virol..

[47] Cheng H.J., Lei H.Y., Lin C.F., Luo Y.H., Wan S.W., Liu H.S., Yeh T.M., Lin Y.S. (2009). Anti-dengue Virus Nonstructural Protein 1 Antibodies Recognize Protein Disulfide Isomerase on Platelets and Inhibit Platelet Aggregation. Mol. Immunol..

[48] Chuang Y.C., Lei H.Y., Lin Y.S., Liu H.S., Wu H.L., Yeh T.M. (2011). Dengue Virus-Induced Autoantibodies Bind to Plasminogen and Enhance Its Activation. J. Immunol..

[49] Chuang Y.C., Lin Y.S., Liu H.S., Wang J.R., Yeh T.M. (2013). Antibodies Against Thrombin in Dengue Patients Contain Both Anti-thrombotic and Pro-fibrinolytic Activities. Thromb. Haemost..

[50] Smith J.L., Flavivirus N.S. (2022). Flavivirus NS1: Structure and Function of an Enigmatic Virulence Factor. FASEB J..

[51] Poungpair O., Bangphoomi K., Chaowalit P., Sawasdee N., Saokaew N., Choowongkomon K., Chaicumpa W., Yenchitsomanus P.T. (2014). Generation of Human Single-Chain Variable Fragment Antibodies Specific to Dengue Virus Non-structural Protein 1 That Interfere with the Virus Infectious Cycle. Mabs.

[52] Pan P., Li G., Shen M., Yu Z., Ge W., Lao Z., Fan Y., Chen K., Ding Z., Wang W. (2021). DENV NS1 and MMP-9 Cooperate to Induce Vascular Leakage by Altering Endothelial Cell Adhesion and Tight Junction. PLoS Pathog..

[53] Chen J., Ng M.M., Chu J.J. (2015). Activation of TLR2 and TLR6 by Dengue NS1 Protein and Its Implications in the Immunopathogenesis of Dengue Virus Infection. PLoS Pathog..

[54] Chu Y.T., Wan S.W., Chang Y.C., Lee C.K., Wu-Hsieh B.A., Anderson R., Lin Y.S. (2017). Antibodies Against Nonstructural Protein 1 Protect Mice from Dengue Virus-Induced Mast Cell Activation. Lab. Investig..

[55] Patro A.R.K., Mohanty S., Prusty B.K., Singh D.K., Gaikwad S., Saswat T., Chattopadhyay S., Das B.K., Tripathy R., Ravindran B. (2019). Cytokine Signature Associated with Disease Severity in Dengue. Viruses.

[56] Rathakrishnan A., Wang S.M., Hu Y., Khan A.M., Ponnampalavanar S., Lum L.C., Manikam R., Sekaran S.D. (2012). Cytokine Expression Profile of Dengue Patients at Different Phases of Illness. PLoS ONE.

[57] Deshmane S.L., Kremlev S., Amini S., Sawaya B.E. (2009). Monocyte Chemoattractant Protein-1 (MCP-1): An Overview. J. Interferon Cytokine Res..

[58] Sierra B., Perez A.B., Vogt K., Garcia G., Schmolke K., Aguirre E., Alvarez M., Volk H.D., Guzman M.G. (2010). MCP-1 and MIP-1Alpha Expression in a Model Resembling Early Immune Response to Dengue. Cytokine.

[59] Kelley J.F., Kaufusi P.H., Nerurkar V.R. (2012). Dengue Hemorrhagic Fever-Associated Immunomediators Induced via Maturation of Dengue Virus Nonstructural 4B Protein in Monocytes Modulate Endothelial Cell Adhesion Molecules and Human Microvascular Endothelial Cells Permeability. Virology.

[60] Soe H.J., Khan A.M., Manikam R., Samudi Raju C., Vanhoutte P., Sekaran S.D. (2017). High Dengue Virus Load Differentially Modulates Human Microvascular Endothelial Barrier Function During Early Infection. J. Gen. Virol..

[61] Talavera D., Castillo A.M., Dominguez M.C., Gutierrez A.E., Meza I. (2004). IL8 Release, Tight Junction and Cytoskeleton Dynamic Reorganization Conducive to Permeability Increase Are Induced by Dengue Virus Infection of Microvascular Endothelial Monolayers. J. Gen. Virol..

[62] Chuang Y.C., Lei H.Y., Liu H.S., Lin Y.S., Fu T.F., Yeh T.M. (2011). Macrophage Migration Inhibitory Factor Induced by Dengue Virus Infection Increases Vascular Permeability. Cytokine.

[63] Glasner D.R., Ratnasiri K., Puerta-Guardo H., Espinosa D.A., Beatty P.R., Harris E. (2017). Dengue Virus NS1 Cytokine-Independent Vascular Leak Is Dependent on Endothelial Glycocalyx Components. PLoS Pathog..

[64] Pries A.R., Kuebler W.M. (2006). Normal Endothelium. Handb. Exp. Pharmacol..

[65] Page A.V., Liles W.C. (2013). Biomarkers of Endothelial Activation/Dysfunction in Infectious Diseases. Virulence.

[66] Schlesinger J.J., Brandriss M.W., Walsh E.E. (1987). Protection of Mice Against Dengue 2 Virus Encephalitis by Immunization with the Dengue 2 Virus Non-structural Glycoprotein NS1. J. Gen. Virol..

[67] Modhiran N., Song H., Liu L., Bletchly C., Brillault L., Amarilla A.A., Xu X., Qi J., Chai Y., Cheung S.T.M. (2021). A Broadly Protective Antibody That Targets the Flavivirus NS1 Protein. Science.

[68] Biering S.B., Akey D.L., Wong M.P., Brown W.C., Lo N.T.N., Puerta-Guardo H., Tramontini Gomes de Sousa F., Wang C., Konwerski J.R., Espinosa D.A. (2021). Structural Basis for Antibody Inhibition of Flavivirus NS1-Triggered Endothelial Dysfunction. Science.

