# Supplementary material for: Potential Protective Effect of Dengue NS1 Human Monoclonal Antibodies against Dengue and Zika Virus Infections
Source: Biomedicines. 2023 Jan 16;11(1):227. doi: 10.3390/biomedicines11010227 (PMC9855337; doi:10.3390/biomedicines11010227)
Supplement: Supplementary file 1 [file biomedicines-11-00227-s001.zip › biomedicines-2071462-supplementary.pdf]

biomedicines

MDPI

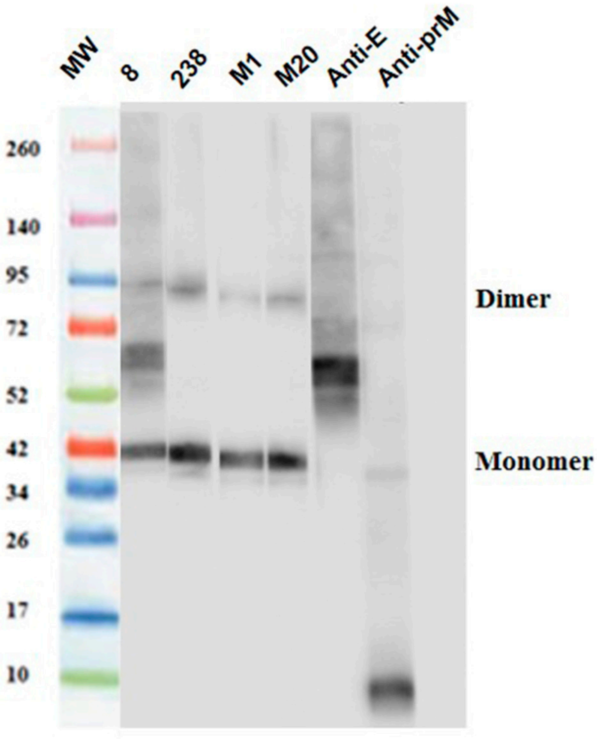

Figure S1. Recognition of native NS1 protein from DENV2 by HuMabs using western blot. DENV-2 -infected C6/36 cell lysates are harvested and fractionated in 10% SDS-polyacrylamide gel. The blots are incubated with four HuMAbs. Lane 1-8 represent molecular weight protein ladder, HuMAbs no. 8, 238, M1, M20, anti-E HuMAb, anti-prM HuMAb, and DMEM medium, respectively.
